# Advancing the Rehabilitation Sciences

**DOI:** 10.3389/fresc.2020.617749

**Published:** 2021-02-10

**Authors:** Gerold Stucki

**Affiliations:** ^1^Department of Health Sciences and Medicine, University of Lucerne, Lucerne, Switzerland; ^2^Center for Rehabilitation in Global Health Systems, University of Lucerne, Lucerne, Switzerland; ^3^Swiss Paraplegic Research, Nottwil, Switzerland

**Keywords:** inclusion, disability, health systems, International Classification of Functioning, Disability and Health, functioning, rehabilitation, rehabilitation sciences

Together with prevention and promotion, cure and palliation, rehabilitation is one of the five health strategies recognized by the World Health Organization (WHO) (1, 2). However, due to population aging and the high prevalence of non-communicable diseases, rehabilitation may become the key health strategy of the 21st century (2, 3). Moreover, as any individual may require and benefit from rehabilitation over the life-course, rehabilitation is an important public health strategy as well.

The need for rehabilitation arises when a person experiences a limitation in functioning (4, 5) associated with a health condition, acute or chronic, that leads to the experience of disability on a short-term or life-long basis (6). To address these needs, health systems should offer rehabilitation services across the care continuum as well as the life span. A wide range of rehabilitation service types (7) and clinical assessment schedules (8) ensure that rehabilitation services can be tailored to a person's needs against the backdrop of their living situation and their country's health system. Rehabilitation may be provided by health professionals who are not specialized in rehabilitation, for example in acute or primary care (9), as well as by health professionals specialized in rehabilitation and working in multi-professional rehabilitation teams, for example in dedicated rehabilitation facilities.

As was first proposed by the Physical and Rehabilitation Medicine Section of the Union of Medical Specialists in Europe in 2007 (4) and revised in 2011 (5), rehabilitation care is guided by the goal of optimizing function in persons “experiencing or likely to experience disability in interaction with the environment”. The 2011 version has served as the basis for a derived conceptual description of the medical specialty for physical and rehabilitation medicine (10), which was endorsed by the International Society of Physical and Rehabilitation Medicine in 2011. The conceptual description of rehabilitation informed WHO's definition in the World Report on Disability (11) and its current definition of rehabilitation: “comprises a set of interventions designed to reduce disability and to optimize functioning in individuals with health conditions so as to enable them to better interact with their environment” (12, 13).

The notion of functioning, which plays a fundamental role in rehabilitation, comes from WHO's International Classification of Functioning, Disability and Health (ICF) (14), a ground-breaking classification that was welcomed by both rehabilitation practitioners and researchers alike (15, 16). The ICF represents a paradigm shift from a solely biomedical perspective to the perspective of a person's lived experience of health in light of a health condition. With the ICF, rehabilitation can rely for the first time on a universally shared conceptual foundation (17). The ICF also provides an operationalization of health (18) that is essential for rehabilitation research. ICF's focus on the “lived experience of health” is also ideally suited for the “conceptualization, organization and development of human functioning and rehabilitation research” from “cell to society” (19, 20).

The ICF is “inextricably entwined with the emergence of rehabilitation as the key health strategy of the 21st century” (17). The strengthening of rehabilitation in health systems worldwide thus requires the integration of functioning information in health information systems worldwide (21, 22). Moreover, functioning is the third health indicator, after mortality and morbidity (23)—both of which are classified using the International Classification of Diseases (24). Together, these three health indicators make it possible to monitor the performance of the health system. The contribution of rehabilitation to health system performance, and ultimately to societal well-being and welfare (25), relies on data on the third health indicator functioning, and these data are reported using ICF as reference system (26).

The introduction of our current understanding of rehabilitation, with functioning as its fundamental concept, allows us to identify a range of grand challenges to the advancement of rehabilitation sciences. These challenges have informed the architecture of the specialty sections of this journal.

## Human Functioning

Recognizing the fundamental role functioning has in rehabilitation, this journal contains a specialty section dedicated to the topic of human functioning. With this section, the journal aims to contribute to the development of the human functioning sciences. Similar to the interdisciplinary field of neurosciences that has been driving discovery and innovation, the human functioning sciences are an emerging scientific field that integrates a wide range of disciplinary perspectives and methodological approaches (19, 20).

## Strengthening Rehabilitation in Health Systems

Following on WHO's call for action in *Rehabilitation 2030*, we include a specialty section devoted to the challenge of strengthening rehabilitation (12), in line with WHO's focus on enhancing health system and policy research (27). To encourage rehabilitation scientists to engage in health systems and policy research, the journal welcomes submissions of policy briefs that can inform stakeholder dialogues (28, 29).

## Disability, Rehabilitation, and Inclusion

The successful implementation of rehabilitation depends on the collaboration across sectors, including health, education, labor, and social welfare. At the level of the individual person, cross-sectorial rehabilitation supports persons in achieving optimal performance in interaction with their immediate environment. Beyond what cross-sectorial rehabilitation services can offer, optimal performance depends on an explicit effort by societies to provide persons with limitations in functioning with fair opportunities (30) and an accessible built and social environment. The aim of this section, therefore, is to contribute to cross-sectorial research, both from the perspective of beneficiaries of rehabilitation and the perspective of an inclusive society.

## Interventions for Rehabilitation

Goals and targets of interventions in rehabilitation can be comprehensively represented by the conceptual framework of the ICF and its categories (31, 32). The aim of this section is to provide the unique opportunity to publish studies on the efficacy, effectiveness and cost-effectiveness across the full range of interventions relevant for rehabilitation. These include studies on interventions that strive to improve a person's intrinsic health capacity and performance in interaction with the immediate environment, to strengthen the resources of the person or aim to modify the physical and social environment. These also include studies on multi-modal, multi-professional, and multi-disciplinary interventions. Although some of these interventions are also used by other health strategies, it is through the specification of a rehabilitation goal that an intervention becomes a rehabilitation intervention.

## Translational Research in Rehabilitation

The scientific foundation for rehabilitation interventions is based on an in-depth understanding of how they work. At the level of intrinsic health capacity and personal psychological factors, translational research aims to translate our understanding of biological mechanisms, and this knowledge can inform the development of clinical interventions. The motivation to develop new interventions based on novel insights can come from basic science, from clinicians, or both working in concert. At the level of a person's performance in interaction with the physical and social environment, translational research aims to translate our understanding of barriers and facilitators into interventions useful for professionals in the health, educational, labor, and social sectors. Accordingly, the aim of this specialty section is to provide space for the publication of translational research that involves the development of novel interventions based on new insights into intervention mechanisms, or research that advances our understanding of interventions used in rehabilitation practice.

## Rehabilitation for Populations with Specific Health Conditions

The specialty sections focusing on specific health conditions pursue two aims. First, the explicitly broadly-named specialty section on “Medical and Surgical Conditions” provides a unique space for the publication of research across a wide range of medical and surgical health conditions that may benefit from rehabilitation (33). Second, the journal offers specialty sections for areas of rehabilitation that are of increasing importance but have previously received less attention. Currently, this includes the specialty sections on “Pulmonary Rehabilitation” and “Rehabilitation for Musculoskeletal Conditions.”

## Conclusion

The mission of this journal is to tackle the grand challenges facing us in the advancement of the rehabilitation sciences. Each specialty section addresses the key research areas that affect not only how rehabilitation interventions are provided to specific populations, but also the structure of the health system in which these interventions take place. The journal recognizes the salience of fundamental concepts that have shaped, and will continue to shape, the development of rehabilitation into the future. Prominent among these is the notion of human functioning, the ICF concept that captures the aim and objective of rehabilitation, the social importance of rehabilitation intersectorially, and the need to be able to translate innovations into practice in a manner that addresses the lived experience of health. The comprehensive concept of functioning serves as a starting point for the full range of scientific approaches and theories and taking different perspectives, including clinical, biomedical, psychological-behavioral, or socio-humanistic.

Across the specialty sections, the journal encourages the publication of methodological developments. This includes studies that address the challenges of applying various research designs and quantitative as well as qualitative methods for rehabilitation research. Of special interest are the opportunities arising from the use of quasi-experimental study designs (34). In the context of the rehabilitation sciences, these study designs are relevant for health system, services and policy research. They may also provide an alternative to randomized controlled trials when studying the effectiveness and cost-effectiveness of rehabilitation interventions.

In conclusion, to address the grand challenges in rehabilitation sciences, the journal aims to continuously and dynamically develop its specialty sections to reflect emerging scientific advances and author contributions.

## Author Contributions

GS conceptualized and wrote the manuscript with support from Professor Jerome Bickenbach, Melissa Selb, and Susanne Stucki.

## Conflict of Interest

The author declares that the research was conducted in the absence of any commercial or financial relationships that could be construed as a potential conflict of interest.
